# A Novel and Highly Inclusive Quantitative Real-Time RT-PCR Method for the Broad and Efficient Detection of Grapevine Leafroll-Associated Virus 1

**DOI:** 10.3390/plants12040876

**Published:** 2023-02-15

**Authors:** Félix Morán, Antonio Olmos, Miroslav Glasa, Marilia Bueno Da Silva, Varvara Maliogka, Thierry Wetzel, Ana Belén Ruiz-García

**Affiliations:** 1 Centro de Protección Vegetal y Biotecnología, Instituto Valenciano de Investigaciones Agrarias (IVIA), Ctra. Moncada-Náquera km 4.5, Moncada, 46113 Valencia, Spain; 2 Biomedical Research Center of the Slovak Academy of Sciences, Institute of Virology, Dúbravská Cesta 9, 84505 Bratislava, Slovakia; 3 Faculty of Natural Sciences, University of Ss. Cyril and Methodius, Nám. J. Herdu 2, 91701 Trnava, Slovakia; 4 Plant Pathology Laboratory, School of Agriculture, Faculty of Agriculture, Forestry and Natural Environment, Aristotle University of Thessaloniki, 541 24 Thessaloniki, Greece; 5 DLR Rheinpfalz, Institute of Plant Protection, Breitenweg, 71, 67435 Neustadt an der Weinstrasse, Germany; 6 Departamento de Microbiología y Ecología, C/Doctor Moliner 50, Burjasot, 46100 Valencia, Spain

**Keywords:** grapevine, leafroll, virus, HTS, diagnostics

## Abstract

Grapevine (*Vitis vinifera* L.) is one of the most important crops in the world due to its economic and social impact. Like many other crops, grapevine is susceptible to different types of diseases caused by pathogenic microorganisms. Grapevine leafroll-associated virus 1 (GLRaV-1) is a virus associated with grapevine leafroll disease and it is considered at the national and European level as a pathogen that must be absent in propagative plant material. For this reason, the availability of specific, sensitive and reliable detection techniques to ascertain the sanitary status of the plants is of great importance. The objective of this research was the development of a new GLRaV-1 detection method based on a TaqMan quantitative real-time RT-PCR targeted to the coat protein genomic region and including a host internal control in a duplex reaction. To this end, three new GLRaV-1 full genomes were recovered by HTS and aligned with all sequences available in the databases. The method has been validated following EPPO standards and applied for the diagnosis of field plant material and transmission vectors. The new protocol designed has turned out to be highly sensitive as well as much more specific than the current available methods for the detection and absolute quantitation of GLRaV-1 viral titer.

## 1. Introduction

Grapevine (*Vitis vinifera* L.) is one of the most important crops cultivated worldwide. Like other crops, grapevine is threatened by pests and diseases caused by different pathogens that can affect production, challenging the sustainability of viticulture. Grapevines can be infected by 89 different virus species [1,2,3,4], representing the largest cultivated host for viral pathogens. The high complexity of the grapevine virome is due to the coevolution between viruses and *Vitis* species, as well as the long domestication history and global trade of this crop [1]. As a consequence, mixed infections requiring an accurate detection and identification of the pathogen are commonly found in grapevine [5,6].

Among all the viruses described to infect grapevine, 31 virus species have been associated with the four major disease complexes based on the symptomatology: infectious degeneration, leafroll, rugose wood and fleck [7]. Grapevine leafroll disease (GLD) is caused by one of the most economically important viral complexes that includes five virus species that belong to the family *Closteroviridae* [7,8]. Typical symptoms are downward rolling of leaf margins, leaf interveinal reddening in red grapevine cultivars and leaf interveinal chlorosis in white cultivars. The quality and yield of berries are compromised and features such as Brix level, maturation and pigmentation are severely affected [9,10].

Grapevine leafroll-associated virus 1 (GLRaV-1) belongs to the GLD complex and is considered by European regulations as a pathogen that must be absent in propagative plant material. GLRaV-1 is a member of the genus *Ampelovirus* transmitted by mealybugs (*Hemiptera: Pseudococcidae*) and soft scale (*Hemiptera: Coccidae*) insects [11,12,13,14]. *Planococcus citri* is one of the vectors involved in GLRaV-1 transmission for which acquisition and transmission rates have been investigated under controlled conditions [15]. It was selected in this study as the preferred vector for the experiments. The GLRaV-1 genome consists of a positive-sense single-stranded RNA of approximately 18.7 to 18.9 kb in length that is encapsidated into filamentous particles [16]. The genome contains ten open reading frames (ORFs) that encode ten proteins, a papain-like protease/methyl transferase/RNA helicase (ORF1a), the RNA-dependent RNA polymerase (ORF1b), p7 (ORF2), a heat shock protein 70-like protein (ORF3), p55 (ORF4), the coat protein (ORF5), the coat protein divergent 1 (ORF6), the coat protein divergent 2 (ORF7), p21 (ORF8) and p24 (ORF9) [16,17]. GLRaV-1 is broadly distributed worldwide [11,18,19,20,21,22]. In Europe and the Mediterranean basin, different studies have demonstrated the spreading of this virus in the most important producing countries including Italy, France, Spain, Greece, Germany, Switzerland, Slovakia, the Czech Republic, Austria, Croatia, Tunisia and Turkey [11,12,23,24,25,26].

Although traditional GLRaV-1 diagnoses have been based on biological indexing and an enzyme-linked immunosorbent assay (ELISA), the limitations of these techniques, such as an inability to identify the virus species, the long-term and high-cost analysis as well as the low sensitivity and specificity, led GLRaV-1 detection towards conventional or real-time RT-PCR methods [27,28,29,30,31,32,33,34,35,36] using several GLRaV-1 genomic regions as target sequences: HSP70h, the selected target region for most of the reported methods [29,30,31,33,34,35,36]; CP [27,29]; CPd2 [29]; RdRp [32]; and p24 [28,29].

The diversity of GLRaV-1 detection methods is related to the high genetic intraspecies variability of this virus for which the presence of up to eight distinct phylogenetic groups have been described [11,12,28,29,35,37,38]. This genetic diversity has important diagnostic implications and represents a major drawback for GLRaV-1 and GLD control. As for other viruses, increasing knowledge of the GLRaV-1 genome is available at public databases, which needs to be considered in order to develop or update highly specific viral detection methods [39,40].

The objective of this study was to develop a highly inclusive diagnostic method able to detect all GLRaV-1 isolates currently known in both plant material and transmission vectors. The developed method was validated according to the European Plant Protection Organization (EPPO) guidelines [41]. We believe that the implementation of the developed method in certification and integrated pest management programs will contribute to a sustainable improvement in grapevine production systems.

## 2. Results

### 2.1. Molecular Characterization of Three GLRaV-1 near Full-Length Genomic Sequences by High-Throughput Sequencing (HTS)

Three grapevine samples infected by GLRaV-1 from Greece (AUTH63), Spain (Pin1) and Slovakia (SK809) were subjected to RNAseq HTS analysis for the characterization of genetic diversity of GLRaV-1 in these samples. The bioinformatic analysis of these datasets allowed for the recovery of three near full-length GLRaV-1 genomes of 18,714 (AUTH63), 18,634 (Pin1) and 18,597 (SK809) nt that were deposited in GenBank (accession numbers OQ029678, OQ029646 and OQ029645, respectively). Details on the bioinformatic analysis performed are included in Table 1. The sequence similarity between these isolates and the GLRaV-1 reference sequence (NC016509.1) was 90.84% (AUTH63), 81.59% (Pin1) and 90.65% (SK809).

### 2.2. GLRaV-1 Genetic Variability Analysis: Diagnostic Implications

All seven full-length or near full-length GLRaV-1 genomic sequences available in the databases to date (accessed on 22 June 2022) from the USA, Canada, France and the Czech Republic, as well as the three near full-length genomic sequences recovered in this study from Greece, Spain and Slovakia, were aligned in order to infer their global genome similarity and phylogenetic relationships.

**Figure 1:**
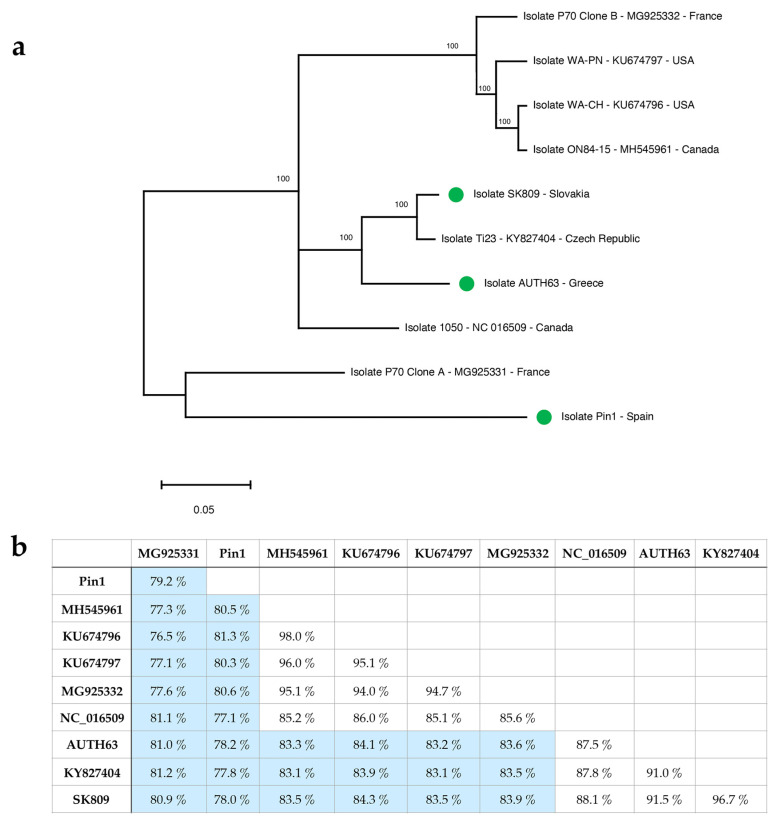
(**a**) Maximum-likelihood phylogenetic tree constructed by MEGA X using the sustitution model GTR + G + I with ten GLRaV-1 complete genomic sequences. Accession numbers and/or isolate names are indicated. The scale bar shows the number of substitutions per site. Bootstrap percentages (1000 resamples) are indicated on the branches. Green dots indicate the new GLRaV-1 genomic sequences obtained in this study. (**b**) Sequence similarity matrix showing the percentages of nucleotide indentity between nearly complete genomes of GLRaV-1 isolates.

The phylogenetic analysis performed with the full-length GLRaV-1 genomic sequences showed the existence of five different phylogenetic groups (Figure 1a), with the French isolate P70 Clone A and the Spanish isolate Pin1 being the two sequences more genetically distant than the others included in this study. According to previous studies [37], the phylogenetic clustering did not seem to be related to the geographic origin. Nevertheless, regarding diagnostic purposes, the high genetic diversity previously reported for this virus species by the analysis of partial sequences is certainly present at the genome level.

In fact, the sequence comparison of full-length GLRaV-1 genomes showed a nucleotide identity between isolates that ranged from 76.5 to 98%, with a mean similarity value of 84.6%, indicating the presence of a high degree of intraspecies variability for this virus (Figure 1b). The high genetic diversity observed at the genome level was in agreement with previous reported data on GLRaV-1 genetic variability based on partial sequences [11,12,28,29,35,37,38] and may lead to important implications for GLRaV-1 detection.

In order to evaluate the impact of this genetic variability on GLRaV-1 diagnosis, primers and probes previously reported in the literature for GLRaV-1 detection on different genomic regions [29,30,32,35,36] were aligned with 659 full-length and partial sequences, including GLRaV-1 sequences available in the databases (accessed on 22 June 2022) as well as the three near full-length sequences recovered in this study. Thus, the presence, frequency and position of primers/probes mismatches were evaluated.

**Figure 2:**
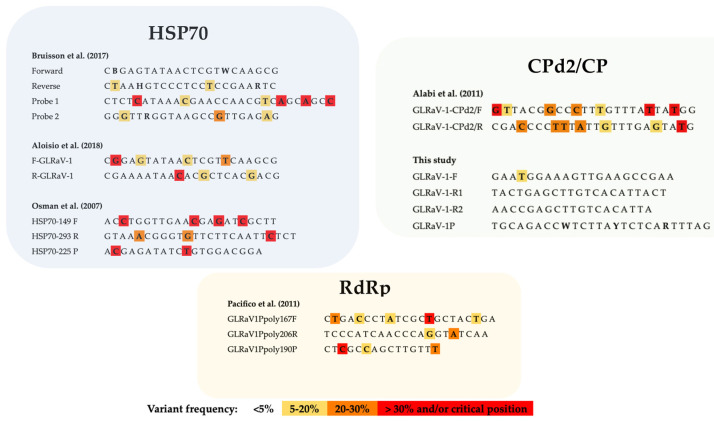
Frequency of primers/probes mismatches in GLRaV-1 detection methods [29,30,35,36]. A score-based color code was created to visualize the mismatches, showing the variant frequency in each position: nucleotides are nonmarked (variant frequency < 5%), yellow (5–20%), orange (20–30%) or red (>30%). Nucleotides located in the four first positions from the 3′ end of the sequence are also colored in red.

This in silico analysis showed the occurrence of a high number of mismatches, ranging from 2 to 7, whereby some of them were also present with variant frequency values higher than 30%. In addition, mismatches located in critical positions close to the 3′ end of the primer, which might compromise primer binding to the target sequence, were found for at least some GLRaV-1 isolates (Figure 2). These two circumstances were observed for most of the previously reported detection protocols which would therefore exhibit important limitations for the inclusive detection of GLRaV-1 intraspecies diversity currently known.

### 2.3. Design of New and Highly Specific GLRaV-1 Primers and Probe

With the aim of reducing the false negative detection rate inferred by the in silico analysis performed on the currently available GLRaV-1 detection methods, the alignment of the 659 sequences described above was used for the design of new primers/probe potentially able to detect all GLRaV-1 diversity known to date. The genomic region encoding for the CP, for which a total of 254 sequences were available, was selected as the target sequence for the design based on the lower level of genetic variability found. Three primers, one forward primer and two reverse primers (GLRaV-1-F, GLRaV-1-R1 and GLRaV-1-R2) and a probe (GLRaV-1P) were designed to target a sequence of 186 bp in the CP gene.

The presence, frequency and position of the mismatches in the primers and probe here designed were evaluated and compared with those detected in the previously reported methods (Figure 2). This in silico comparison indicated a much better scenario for the primers and probe designed in this study that only showed the presence of one mismatch in the forward primer with a variant frequency value of 17.1%. Moreover, the mispairing position was located close to the 5’ end of the primer and conserved the critical 3′ positions among almost all GLRaV-1 isolates known. No mismatches with a variant frequency higher than 5% were found in the designed probe (Figure 2).

### 2.4. Detection of GLRaV-1 by Duplex Quantitative Real-Time RT-PCR

A duplex real-time RT-PCR method was composed using the newly designed GLRaV-1 primers and probe as well as primers and probes amplifying an internal grapevine control, the phosphoenolpyruvate carboxylase (PEP) gene [42]. This internal control will allow the identification of putative false negative results, thus increasing the diagnostic sensitivity of the technique.

The ability of the method to detect GLRaV-1 was evaluated by analyzing 65 positive samples from seven different geographic origins and 16 different grape varieties. All the positive controls were also tested by two other methods, one of the most frequently used real-time RT-PCR methods [30] and a conventional RT-PCR technique [29] previously reported.

GLRaV-1 was successfully detected in all positive samples tested using the new real-time RT-PCR method designed in this study with Ct values ranging from 14.9 to 38.0 (Supplementary Table S1) and the Sanger sequencing confirmation of amplicons. All the samples also gave a positive amplification signal for the plant internal control. No signal was detected for the plants obtained from the meristem tip culture sanitized grapevine cultivars used as a negative control in this study.

These results demonstrated the high detection inclusivity exhibited by our technique. The method described by Osman et al. [30] was only able to detect 35 out of the 65 positive samples (53.8%), whereas the conventional protocol [29] could only detect 23.1% of them.

### 2.5. Validation of the Designed GLRaV-1 Real-Time RT-PCR

#### 2.5.1. Validation of the Plant Internal Control

**Figure 3:**
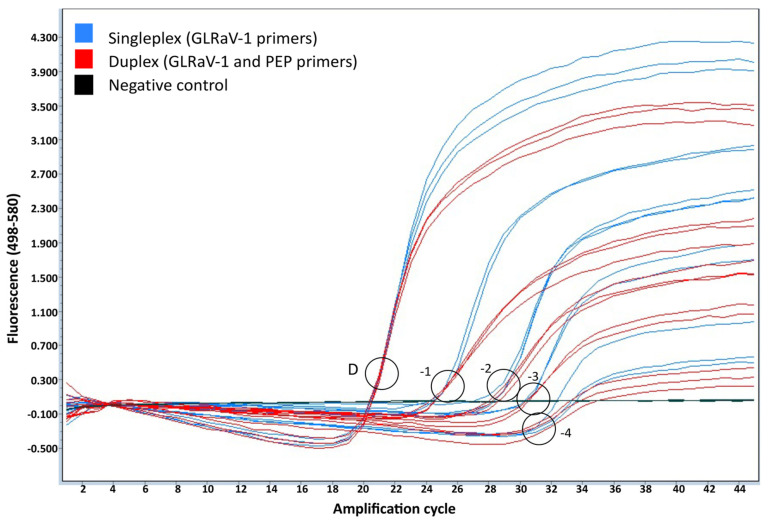
Validation of a grapevine internal control in a GLRaV-1 duplex real-time RT-PCR assay. Three independent replicates of a GLRaV-1 infected plant extract diluted with healthy plant extracts were assayed. GLRaV-1 amplification plots are shown for singleplex (blue) and duplex (red) reactions. A negative control (black) is included. PEP: phosphoenolpyruvate carboxylase; D: undiluted plant extract. −1:10^−1^ dilution. −2:10^−2^ dilution. −3:10^−3^ dilution. −4:10^−4^ dilution.

To exclude the possibility of the grapevine internal control amplification interfering with GLRaV-1 detection in the duplex RT-PCR reaction, the GLRaV-1 amplification signal obtained in the singleplex reaction (using only GLRaV-1 primers and probe) and the one obtained in the duplex reaction (using both virus and plant internal control primers and probe) were compared. Three independent replicates of four ten-fold serial dilutions (from 10^−1^ to 10^−4^ dilution) of GLRaV-1-infected plant extracts diluted in healthy plant extracts were analyzed in both singleplex and duplex conditions. The results of this analysis showed that GLRaV-1 detection was not affected by the introduction of an internal control in a duplex reaction (Figure 3). The use of an internal control in a duplex reaction allows for the identification of putative false negative results, thus increasing the diagnostic sensitivity of the technique.

#### 2.5.2. Technical Sensitivity and Absolute Quantification

**Figure 4:**
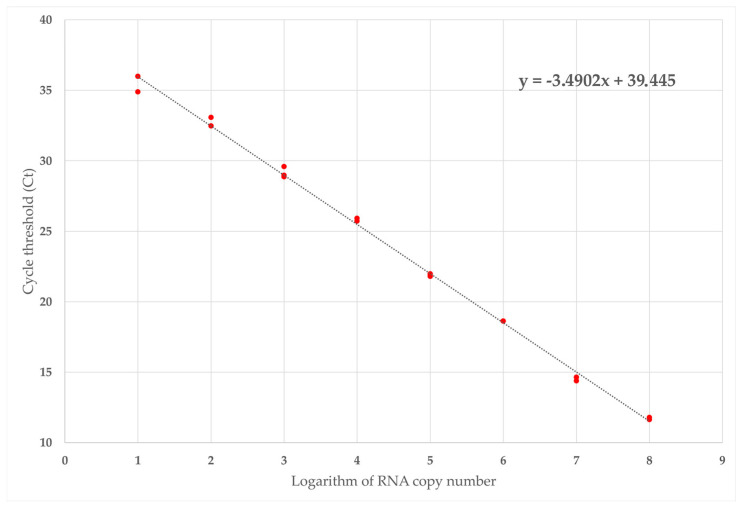
Absolute GLRaV-1 quantitation standard curve. Cycle threshold (Ct) values obtained for three replicates of ten-fold serial dilutions of GLRaV-1 control transcripts are plotted. The mathematical equation of the standard curve used for quantification and the coefficient of correlation (R^2^) are indicated.

The absolute quantitation of GLRaV-1 was performed using known quantities of in vitro transcripts containing a CP sequence targeted by the real-time RT-PCR. A standard curve was obtained using three replicates of serial dilutions ranging from 4.4 × 10^8^ to 44 target copies (Table 2, Figure 4). The standard curve showed a slope of −3.49 which allowed us to calculate an amplification efficiency of 93.43% with a correlation coefficient (R^2^) of 0.9973. Therefore, the GLRaV-1 detection method designed in this study was able to detect up to 44 copies of viral targets. The technical sensitivity in the plant material showed a detection limit of 67 viral copies (Supplementary Table S2).

#### 2.5.3. Analytical Specificity and Selectivity

The analytical specificity of the technique was evaluated considering both inclusivity and exclusivity. Inclusivity was evaluated by testing different GLRaV-1 isolates from different geographic origins. All positive samples tested, representing the GLRaV-1 genetic diversity, were successfully detected by the method herein developed. Exclusivity was evaluated by testing eight GLRaV-1-free grapevine plants infected by several common grapevine viruses, as determined by HTS (Table 3). None of these samples gave a false positive signal when tested by our method (data not shown).

The selectivity of our method was evaluated by testing GLRaV-1 isolates infecting 16 different grapevine cultivars. No effect of the matrix on the testing performance was observed in any case.

#### 2.5.4. Repeatability and Reproducibility

Repeatability and reproducibility were evaluated by analyzing seven GLRaV-1-infected samples with a low relative concentration. Nine technical replicates of each sample were tested using three different types of equipment (three replicates per thermal cycler) on different days and by two different operators. All assays gave a positive result. The mean Ct values, standard deviations and coefficient of variation obtained are shown in Table 4. The coefficient of variation observed between the replicates tested in the same thermal cycler ranged from 1.6 to 8.5%. The coefficient of variation between the nine replicates independent of the equipment used did not exceed 12%. These results indicate a high level of consistency in the performance of the technique.

### 2.6. Performance of the New Real-Time RT-PCR Method for GLRaV-1 Diagnosis in Field Samples

The method here developed was implemented for GLRaV-1 detection in field samples. A total of 241 samples from several random surveys from different Spanish grapevine-growing regions (D.O. Priorato, D.O. Manchuela and D.O. Utiel-Requena) that had previously tested negative by the RT-PCRs reported by Osman et al. [30] and Alabi et al. [29] were tested (Table 5). Interestingly, 24 of these samples tested positive for GLRaV-1, supporting the higher inclusivity of the designed protocol with respect to the previously available techniques. The plant internal control tested positive in 234 samples out of 241, thus identifying seven putative false negative results.

### 2.7. Quantitative Detection of GLRaV-1 in Planococcus Citri

To assess the capacity of the technique to detect GLRaV-1 in transmission vectors, *Planococcus citri* mealybugs fed GLRaV-1-positive plants were analyzed with the quantitative real-time RT-PCR developed in this study. GLRaV-1 was identified in a total of six mealybugs from two independent acquisition experiments. The viral titer determined in the insects ranged from 45,162 to 209,530 viral targets. The mealybugs fed on the GLRaV-1 free grapevines tested negative. These results show the ability of the method to detect GLRaV-1 not only in plant material but also in transmission vectors.

## 3. Discussion

The specific and reliable detection of viruses in plant material and transmission vectors is an essential tool in the management and control of plant viral diseases. GLRaV-1 belongs to the GLD complex that causes a significant impact in grapevine production, and therefore is considered one of the most important grapevine pathogens regulated by European legislation [43].

Despite being a broadly distributed [11,25,26] and a well-known virus systematically tested by plant health services worldwide [28,30,32,44,45,46], GLRaV-1 diagnosis is currently far from trivial [47]. The high degree of the intraspecies variability of the virus represents a challenge for the development of highly inclusive diagnostic methods with the potential to detect its genetic diversity [11,12,28,29,35,37,38]. Moreover, our knowledge of this viral genetic diversity is continuously increasing as new genomic information is being incorporated into the databases. Especially in the last years, with the advent of HTS technologies, the resources of viral genome sequence data and related information have been continuously increasing [48,49,50,51]. The aim of this study was the development of a new real-time quantitative RT-PCR method having the required inclusivity to be able to detect all the genetic variability known to date for this virus.

For this purpose, we studied GLRaV-1 genetic diversity using both sequence information available in the databases and genomic data generated in this study by HTS. To date, only seven GLRaV-1 full-length genomes from the USA, France, the Czech Republic and Canada were publicly available. In this study, we obtained and incorporated three new nearly full genome sequences from different geographic origins, Greece, Spain and Slovakia, into GenBank, thus increasing in a significant way the available GLRaV-1 genome information. A sequence comparison and phylogenetic analysis have indicated that the high genetic diversity previously reported at certain GLRaV-1 genomic regions is indeed present. The current GLRaV-1 genomic knowledge has been used to design a new real-time quantitative RT-PCR method for the detection of this virus. This protocol has been validated according to EPPO guidelines in order to guarantee the reliable use of this technique by plant sanitary services [41].

This method was composed as a duplex reaction including the amplification of a plant internal control, the grapevine PEP gene, in order to identify putative false negative results. The reduction in the negative results of the method provides an increase in the diagnostic sensitivity of the technique, thus enhancing the reliability of the protocol. Technical sensitivity has also been shown to be very high, as it is the method that is able to detect as little as 67 viral targets in plant material.

Interestingly, GLRaV-1 detection by this new method is not only very sensitive but also highly specific. Specificity is related to inclusivity, which is the performance of the test with a range of target organisms covering diversity, different geographical origins and hosts, and exclusivity, which is the absence of a reaction with a range of nontargets closely related to or commonly present in the matrix. This new protocol has been shown to be by far much more inclusive than the methods with which it was compared in silico and/or in vitro. Regarding exclusivity, any false positive result was obtained by this technique when tested on samples infected by common grapevine viruses.

In addition, the method here developed was successfully applied for the detection of GLRaV-1 in both plant material from field samples and transmission vectors, allowing for the absolute quantitation of the viral titer as well.

In conclusion, in this study, we report the design of a new and highly inclusive quantitative real-time RT-PCR method for the broad and efficient detection of GLRaV-1. The method, which has been validated following EPPO standards, can be applied to certification and integrated pest management programs to improve sustainable grapevine production worldwide.

## 4. Material and Methods

### 4.1. Plant and Insect Material

A total of 65 GLRaV-1-positive samples from different geographic origins, Spain, Switzerland, Slovakia, Tunisia, Thailand, Greece and Germany, and different varieties (Tempranillo, Bobal, Pinot Noir, Rèze, Räuschling, Veltliner, Muller-Thurgau, Gewurztraminer, Marsaoui, Roditis, Vertzami, Mavrothiriko, Geisenheim 26, Chardonnay, Pinot Blanc and Riesling) were used for the validation of the new detection method. Grapevine plants that originated from three meristem tip culture sanitized grapevine cultivars were used as negative controls.

In addition, 241 field samples collected in several random surveys from different Spanish grapevine-growing regions (D.O. Priorato, D.O. Manchuela and D.O. Utiel-Requena) that had previously tested negative using previously reported GLRaV-1 detection methods [29,30] were analyzed using the new method developed in this study.

*Planococcus citri* mealybugs were kindly provided by Dr. Francisco Beitia (IVIA). The colonies were maintained in lemon fruits for two months at room temperature. The absence of GLRaV-1 was confirmed in 3 pools of 5 insects after one month and just before the acquisition. For acquisition, 10–20 mealybugs were placed on grapevine leaves from GLRaV-1-infected plants and were incubated at 28 °C and 60% humidity in the plant growth chamber (Sanyo MLR-350H) with a 12/12 h light cycle. After acquisition during 48 h, 6 insects were individually analyzed. The mealybugs fed on healthy grapevine leaves were used as negative controls.

### 4.2. Sample Preparation and RNA Purification

Stem and/or leaf tissues from each plant sample were placed in individual plastic bags (Bioreba, Reinach, Switzerland) with an extraction buffer (PBS containing 0.2% of diethyldithiocarbamate and 2% of polyvinylpyrrolidone-10) in a ratio of 1:5 (w:v). The samples were grinded with Homex 6 (Bioreba, Reinach, Switzerland).

Total RNA was purified from 200 μL of plant extract using a commercial Plant/Fungi Total RNA Purification Kit (Norgen Biotek Corporation, Thorold, ON, Canada) following the manufacturer’s instructions. The RNA was eluted in a total volume of 50 μL and quantified using a DeNovix DS-11 spectrophotometer (DeNovix Inc., Wilmington, DE, USA). All RNA purifications were stored at −80 °C until subsequent analysis.

The RNA from mealybugs was purified using a Plant/Fungi Total RNA Purification Kit (Norgen Biotek Corporation, Thorold, ON, Canada) with slight modifications. Briefly, the mealybugs were placed in tubes containing 250 μL of a lysis buffer and a similar volume of 212–300 μm glass beads (Sigma-Aldrich, Burlington, MA, USA). The tissue was homogenized, shaken in a vortex for 2 min and centrifuged for 5 min at 10,000× *g*. The supernatant was transferred to a fresh tube and the extraction procedure was followed as indicated by the manufacturer.

### 4.3. HTS Analysis and Genome Recovery

The HTS raw data were analyzed using CLC Genomics Workbench 10.1.1 (Qiagen Bioinformatics, Hilden, Germany) and Geneious Prime 2022 software (Biomatters Ltd., Auckland, New Zealand). RNA quality control, library construction and HTS sequencing in a NextSeq 500 platform (paired 2 × 150 nt) were performed at Macrogen Inc. (Seoul, Republic of Korea). Complementary DNA was synthesized from each RNA extraction for library preparation using the TruSeq Stranded Total RNA LT Sample Prep Kit (Plant). The library protocol preparation used for it was the TruSeq Stranded Total RNA Sample Prep Guide, Part #15031048 Rev. 

Raw reads were subjected to the trimming of adapters and quality control using CLC Genomics Workbench 10.1.1 (Qiagen Bioinformatics, Hilden, Germany). Host genome subtraction was performed by mapping the reads against the reference sequences GCF_000003745.3_12X, FM179380 and DQ424856 corresponding to the grapevine’s genome, mitochondria and chloroplast, respectively.

Grapevine-unrelated reads were de novo assembled using the CLC Genomics Workbench 10.1.1 (Qiagen Bioinformatics, Hilden, Germany). Generated contigs higher than 200 nt were analyzed by BLASTN/BLASTX (e-value < 10^−4^). For full-length genome recovery, GLRaV-1 related contigs were exported to Geneious Prime 2022 software (Biomatters Ltd., Auckland, New Zealand). The contigs were extended by mapping the reads against the contigs. 

### 4.4. GLRaV-1 Detection Using Previously Reported RT-PCR Protocols

The 65 GLRaV-1-positive samples were tested by using two previously described protocols, a conventional RT-PCR [29] and one real-time RT-PCR [30]. The conventional RT-PCR [29] was performed in the Veriti 96 Well thermal cycler (Applied Biosystems, Foster City, CA, USA). The reaction was performed in a total volume of 25 μL using the master mix AgPath-ID™ (Ambion Inc., Austin, TX, USA) containing 500 nM of each primer and 50 ng of total purified RNA. The amplification conditions consisted of an initial reverse transcription step at 45 °C for 45 min followed by a denaturation step at 95 °C for 10 min and 40 cycles of amplification (30 s at 95 °C, 30 s at 50 °C and 25 s at 60 °C) with a final elongation step at 60 °C for 7 min. The real-time RT-PCR protocol [30] was carried out in a StepOnePlus thermal cycler (Applied Biosystems, Foster City, CA, USA). The reaction was performed in a total volume of 12 μL using a master mix AgPath-ID™ One-Step RT-PCR Kit (Ambion Inc., Austin, TX, USA) containing 500 nM of each primer, 125 nM of TaqMan probe and 50 ng of total purified RNA. The amplification conditions were an initial reverse transcription step at 45 °C for 10 min and a denaturation step at 95 °C for 10 min, followed by 45 cycles of amplification (15 s at 95 °C, 15 s at 49 °C and 45 s at 60 °C).

### 4.5. Phylogenetic Analysis of GLRaV-1 Full-Length Genomes

All full-length GLRaV-1 genomic sequences available in the databases (NC016509, MH545961, KU674796, KU674797, MG925331, MG925332, KY827404) from Canada, the USA, France and the Czech Republic, as well as the three genomic sequences obtained in this study from Greece, Spain and Slovakia (OQ029678, OQ029646 and OQ029645, respectively) were aligned using the ClustaW implemented in MEGA X [52]. A phylogenetic tree was constructed with the maximum likelihood algorithm implemented in MEGA X by applying the General Time Reversible [53] model employing a discrete gamma distribution to model evolutionary rate differences among sites with invariant sites (+G), allowing for some sites to be evolutionary invariable (+I) and 1000 bootstrap replicates.

### 4.6. In Silico Evaluation of GLRaV-1 Detection Methods Specificity

A total of 659 GLRaV-1 sequences including full and partial genomes registered in NCBI (accessed on 22nd June 2022) as well as three near full-length sequences recovered in this study were aligned using the algorithm Geneious included in Geneious Prime 2022 software (Biomatters Ltd., Auckland, New Zealand).

The sequence mismatching of several GLRaV-1 primers and probes previously reported in the literature was evaluated and classified in a variant frequency score scale: <5%; 5–20%; 20–30%; >30%. The position of the mismatches in the sequence was also analyzed. Those nucleotides located less than four residues away from the 3′ end of the primers were considered as placed in critical positions.

### 4.7. GLRaV-1-Specific Primers and Probe Design

New GLRaV-1-specific primers and probes were designed based on the alignment mentioned above: a forward primer, GLRaV-1-F, 5′-GAATGGAAAGTTGAAGCCGAA-3′; two reverse primers, GLRaV-1-R1, 5′-TACTGAGCTTGTCACATTACT-3′ and GLRaV-1-R2, 5′-AACCGAGCTTGTCACATTA-3′; and a probe, GLRaV-1P, 5′-6-FAM-TGCAGACCWTCTTAYTCTCARTTTAG-ZNA-4-BHQ-1-3′. The primers and probe were purchased from metabion international AG (Martinsried, Bavaria, Germany).

### 4.8. TaqMan Quantitative Real-Time GLRaV-1 RT-PCR Detection Method

The real-time RT-PCR was performed as a duplex reaction using the GLRaV-1-specific primers and probe detailed above, as well as a set of primers and probe amplifying the grapevine gene phosphoenolpyruvate carboxylase (PEP) used as a plant internal control, PEP-F1 (5′-GCCTCCTCCTCCAGATTGCT-3′), PEP-R1 (5′-AGGCTTGCTTGATTCCATTATCTCTTTCG-3′) and PEP-probe (5′-Cy5-CGACCCATACTTGAAACAGAGACTCCGGC-ZNA-BHQ2-3′) [42].

RT-PCR assays were carried out in a LightCycler 480 thermocycler (Roche, Basel, Switzerland), QuantStudio 3 real-time PCR system (Applied Biosystems, Foster City, CA, USA) and StepOne Plus thermal cycler (Applied Biosystems, Foster City, CA, USA) using the Prime Script TM One Step RT-PCR Kit (Takara Bio Inc., Kusatsu, Japan). The master mix contained 1.2 μM of each GLRaV-1 primer (GLRaV-1-F, GLRaV-1-R1 and GLRaV-1-R2), 100 nM of each internal control primer (PEP-F1 and PEP-R1), 200 nM of probe GLRaV-1P and 50 nM of the PEP-probe. The reaction mixture was carried out in a total volume of 20 μL containing 50 ng of RNA template. The amplification conditions consisted of 45 °C for 10 min, 95 °C for 10 min and 45 cycles of amplification (15 s at 95 °C, 1 min at 60 °C). The default threshold set by the machine was slightly adjusted above the noise to the linear part of the growth curve at its narrowest point, according to the manufacturer.

### 4.9. Absolute Quantitation and Evaluation of Sensititvity

For the generation of real-time qPCR standard curves, the CP fragment targeted by the real-time RT-PCR was amplified by a conventional RT-PCR from the positive sample LR-1 using the GLRaV-1 primers designed in this study. The reaction mixture was composed of 1 µM of each of the GLRaV-1 primers, 5U AMV Reverse Transcriptase (Promega Corporation, Madison, WI, USA), 2.5U GoTaq^®^ G2 Flexi DNA Polymerase (Promega Corporation, Madison, WI, USA) and 50 ng of RNA template in a final volume of 25 μL. The amplification conditions consisted of a reverse transcription step at 42 °C for 45 min and a denaturation step at 95 °C for 2 min, followed by 40 cycles of amplification (15 s at 95 °C, 30 s at 55 °C and 20 s at 72 °C) and a final step of 10 min at 72 °C.

The PCR product (186 bp) was purified using a commercial mi-PCR Purification Kit (metabion international AG, Martinsried, Germany) and cloned into a pGEM^®^-T Easy Vector (Promega Corporation, Madison, WI, USA) following the manufacturer’s instructions and cloned in *Escherichia coli* XL1-Blue cells. The transformed colonies were selected by ampicillin resistance on LB plates containing 100 µg/mL of ampicillin.

A plasmid extraction was performed using a PureYieldTM Plasmid Miniprep System (Promega Corporation, Madison, WI, USA) following the manufacturer’s instructions and was quantified with a DeNovix DS-11 spectrophotometer (DeNovix Inc, Wilmington, DE, USA). The plasmid was then linearized using *Sal I* and an in vitro transcription carried out using T7 RNA polymerase (Takara Bio Inc., Kasatsu, Japan) following the manufacturer’s instructions. Briefly, 500 ng of a linearized plasmid was used as a template for the in vitro RNA transcription. The reaction mixture containing 800 nM NTPs and 50 U T7 RNA pol, in a total volume of 20 µL, and was incubated at 42 °C for 90 min. The solution was then subjected to a DNA digestion with RQ1 RNase-free DNase (Promega Corporation, Madison, WI, USA) for 30′ at 37 °C following the manufacturer’s instructions. The purification of RNA was carried using a Plant/Fungi Total RNA Purification Kit spin column and quantified.

Using the following formula, the number of transcripts was calculated considering the average weight of ribonucleotides and the total base pairs of the transcript: ssRNA = (μg of ssRNA × 10^6^)/(340 × *N*b). To estimate the number of transcripts, Avogadro’s constant [54] was used (6.023 × 10^23^ molecules/mol). Three replicates of serial dilutions from 4.4 × 10^8^ to 44 plasmid copies were used to generate the standard curve. The amplification efficiency of the RT-PCR was evaluated based on the standard curve slope using amplification efficiency = [10 (−1/slope)] − 1 [55].

Sensitivity in the plant material was evaluated by analyzing ten-fold serial dilutions of naturally infected plant material diluted in healthy plant extracts. The absolute quantification of three replicates of each dilution was performed using the generated standard curves, and the viral titer was determined.

## Figures and Tables

**Table 1:** HTS bioinformatic analysis of GLRaV-1 infected grapevines. Details of reads/contigs numbers at each analysis step are indicated.

	Sample		
Number	PIN1	AUTH63	SK809
Total raw reads	60,630,138	62,528,966	1,471,284
Reads after QC	60,597,198	40,061,946	1,269,123
Reads after grapevine genome subtraction	8,642,008	827,368	8360
GLRaV-1 related contigs	1	4	1

**Table 2:** GLRaV-1 real-time RT-PCR quantitation range. Ct values for different amounts of GLRaV-1 CP RNA transcripts are indicated.

Number of Target Copies	Ct Value Replicate 1	Ct Value Replicate 2	Ct Value Replicate 3	Average ± SD
4.4 × 10^8^	12.28	11.55	11.69	11.84 ± 0.39
4.4 × 10^7^	14.55	14.01	14.29	14.28 ± 0.27
4.4 × 10^6^	19.30	18.53	18.53	18.79 ± 0.44
4.4 × 10^5^	21.89	21.71	21.78	21.79 ± 0.09
4.4 × 10^4^	25.91	25.82	25.63	25.79 ± 0.14
4.4 × 10^3^	28.77	28.88	29.50	29.05 ± 0.39
4.4 × 10^2^	32.98	32.37	33.46	32.94 ± 0.55
4.4 × 10	34.80	35.90	35.59	35.43 ± 0.57

**Table 3:** Virome analysis of GLRaV-1 free grapevine samples determined by HTS.

Sample	Origin	Virome ^(1)^
33.17	Spain	GLRaV-3; GRSPaV; GRVFV; GAMaV; GFkV; GVA; GYSVd-1
33.24	Spain	GLRaV-4; GLRaV-3; GYSVd-1
33.28	Spain	GLRaV-3; GRSPaV; GRVFV; GAMaV; GVA; GYSVd-1
33.35	Spain	GLRaV-4; GLRaV-3; GYSVd-1; GRVFV
33.47	Spain	GLRaV-3; GRSPaV; GRVFV; GYSVd-1
30T	Spain	GLRaV-3; GLRaV-2; GLRaV-4; GRSPaV; GVA; GFkV; GPGV; GYSVd-1
U24	Spain	GLRaV-3; GLRaV-4; GRSPaV; HSVd; GYSVd-1
29.9	Greece	GLRaV-2; GLRaV-4; GRLDaV; GRVFV; HSVd; GYSVd-1

^(1)^ GLRaV-3: grapevine leafroll-associated virus 3; GRSPaV: grapevine rupestris stem pitting-associated virus; GRVFV: grapevine rupestris vein feathering virus; GAMaV: grapevine asteroid mosaic virus; GFkV: grapevine fleck virus; GVA: grapevine virus A; GYSVd-1: grapevine yellow speckle viroid 1; GLRaV-2: grapevine leafroll-associated virus 2; GLRaV-4: grapevine leafroll-associated virus 4; HSVd: hop stunt viroid; GRLDaV: grapevine roditis leaf discoloration associated virus; GPGV: grapevine Pinot gris virus.

**Table 4:** Evaluation of repeatability and reproducibility of the quantitative real-time RT-PCR. Mean Ct values, standard deviation and coefficient of variation (CV) between replicates performed in the same thermal cycler and between all replicates are shown.

Samples	StepOne Plus		QuantStudio		Roche 480		All Equipments	
	Mean Ct ± SD	CV (%)	Mean Ct ± SD	CV (%)	Mean Ct ± SD	CV (%)	Mean Ct ± SD	CV (%)
91.1	32.34 ± 2.74	8.5	27.15 ± 1.79	6.6	31.37 ± 1.55	5.0	30.29 ± 2.79	9.2
91.2	31.40 ± 1.40	4.5	29.33 ± 0.95	3.3	31.54 ± 0.50	1.6	30.76 ± 1.58	5.1
91.10	33.62 ± 1.01	3.0	32.43 ± 0.58	1.8	32.43 ± 1.71	5.3	32.83 ± 0.68	2.1
91.11	33.68 ± 1.69	5.0	30.61 ± 0.73	2.4	33.84 ± 0.68	2.0	32.71 ± 1.82	5.6
98.16	34.29 ± 0.98	2.9	33.64 ± 0.84	2.5	32.65 ± 0.75	2.3	33.53 ± 0.83	2.5
102.18	24.05 ± 1.91	7.9	21.49 ± 1.53	7.1	26.48 ± 1.71	6.5	24.00 ± 2.50	10.4
102.20	25.64 ± 1.08	4.2	20.48 ± 0.39	1.9	24.50 ± 0.49	2.0	23.54 ± 2.71	11.5

**Table 5:** Results of the survey performed with the new GLRaV-1 detection method.

Origin	Collection Date	Number of Samples	Positive	Negative
Utiel-Requena	2015	48	5 (10.4%)	43 (89.6%)
Priorato	2016	29	0 (0.0%)	29 (100.0%)
Manchuela	2016	26	3 (11.5%)	23 (88.5%)
Utiel-Requena	2019	52	0 (0.0%)	52 (100.0%)
Utiel-Requena	2021	86	16 (18.6%)	70 (81.4%)
Total		241	24 (10.0%)	217 (90.0%)

## Data Availability

All data supporting the results and conclusions of the study are contained within the article. Generated sequences have been submitted to GenBank. HTS reads covering the full-length genomes recovered in this study are available upon request.

## Supplementary Materials

The following supporting information can be downloaded at https://www.mdpi.com/article/10.3390/plants12040876/s1: Table S1: Geographic origin, cultivar and GLRaV-1 testing results by the methods compared in the study; Table S2: Analytical sensitivity evaluation and absolute quantification of ten-fold serial dilutions of GLRaV-1-infected plant extracts. References [29,30,56] are cited in the supplementary materials.
