# Supplementary material for: A Novel and Highly Inclusive Quantitative Real-Time RT-PCR Method for the Broad and Efficient Detection of Grapevine Leafroll-Associated Virus 1
Source: Plants (Basel). 2023 Feb 15;12(4):876. doi: 10.3390/plants12040876 (PMC9962094; doi:10.3390/plants12040876)
Supplement: Supplementary file 1 [file plants-12-00876-s001.zip › Table S1.pdf]

Table S1. Geographic origin, cultivar and GLRaV-1 testing results by the methods compared in the study.

GLRaV-1 positive sample*	Variety	Origin	Conventional RT-PCR (Alabi *et al.*, 2011)	Real-time RT-PCR (Osman *et al.*, 2007) Average CTs ± SE	New real-time RT-PCR protocol (This study) Average CTs ± SE	
					GLRaV-1	PEP
LR-1	Tempranillo	Spain	+	28.8 ± 0.09	25.0 ± 0.13	20.1 ± 0.12
52.1	Marsaoui	Tunisia	-	-	36.8 ± 0.45	21.5 ± 0.21
88.1	Pinot Noir	Switzerland	+	17.7 ± 0.15	17.5 ± 0.30	20.7 ± 0.16
88.2	Rèze		+	17.9 ± 0.12	18.5 ± 0.26	21.8 ± 0.18
88.3	Räuschling		+	18.6 ± 0.22	18.6 ± 0.14	23.9 ± 0.17
35.2	White Malaka	Thailand	+	22.8 ± 0.21	24.9 ± 0.22	21.9 ± 0.45
35.4	White Malaka		+	-	23.7 ± 0.25	20.3 ± 0.14
35.6	Flame Seedless		+	19.8 ± 0.45	18.8 ± 0.19	20.1 ± 0.13
91.1	Räuschling	Slovakia	-	-	28.9 ± 0.32	22.3 ± 0.11
91.2	Veltliner		-	-	29.0 ± 0.18	23.7 ± 0.21
91.3	Veltliner		-	-	17.2 ± 0.13	21.6 ± 0.16
91.4	Veltliner		+	-	18.9 ± 0.14	20.3 ± 0.14
91.5	Veltliner		-	-	18.3 ± 0.05	21.7 ± 0.20
91.6	Veltliner		-	-	34.0 ± 0.41	22.3 ± 0.23
91.7	Veltliner		-	27.4 ± 0.54	33.0 ± 0.36	23.5 ± 0.14
91.8	Muller-Thurgau		-	15.0 ± 0.48	14.9 ± 0.12	24.6 ± 0.21
91.9	Muller-Thurgau		-	-	-	21.0 ± 0.18
91.10	Muller-Thurgau		-	33.0 ± 0.18	31.8 ± 0.45	20.4 ± 0.19
91.11	Muller-Thurgau		-	-	32.8 ± 0.56	25.3 ± 0.16
91.12	Gewurztraminer		+	-	23.3 ± 0.12	24.1 ± 0.15
18.6	Mavrothiriko	Greece	-	-	38.0 ± 0.9	21.3 ± 0.20
19.3	Mavrothiriko		+	19.7 ± 0.16	17.8 ± 0.32	23.1 ± 0.23
19.5	Vertzami		+	-	18.9 ± 0.55	20.4 ± 0.26
19.6	Roditis		-	-	35.3 ± 0.13	21.7 ± 0.12
29.4	Roditis		-	21.4 ± 0.35	17.8 ± 0.78	22.8 ± 0.22

29.6	Roditis		-	19.0 ± 0.11	19.7 ± 0.13	24.1 ± 0.14
29.8	Roditis		-	-	33.2 ± 0.14	22.3 ± 0.31
29.11	Roditis		-	-	30.7 ± 0.65	20.5 ± 0.16
98.1	Roditis		-	-	-	24.7 ± 0.14
98.2	Roditis		-	-	17.7 ± 0.16	21.8 ± 0.16
98.3	Roditis		-	16.7 ± 0.18	16.2 ± 0.32	20.4 ± 0.17
98.5	Roditis		-	-	-	20.7 ± 0.14
98.6	Roditis		-	-	-	21.5 ± 0.23
98.7	Roditis		-	-	-	22.5 ± 0.18
98.8	Roditis		-	19.5 ± 0.16	15.3 ± 0.14	23.7 ± 0.21
98.9	Roditis		-	20.3 ± 0.19	24.0 ± 0.18	22.7 ± 0.19
98.10	Roditis		-	22.4 ± 0.13	22.0 ± 0.13	21.8 ± 0.15
98.11	Roditis		+	20.4 ± 0.09	21.7 ± 0.11	20.9 ± 0.21
98.12	Roditis		-	-	26.5 ± 0.22	21.2 ± 0.13
98.13	Roditis		-	21.6 ± 0.24	20.2 ± 0.17	24.3 ± 0.17
98.14	Roditis		-	-	32.1 ± 0.13	21.6 ± 0.16
98.15	Roditis		-	-	-	25.0 ± 0.23
98.16	Roditis		-	-	30.9 ± 0.45	23.4 ± 0.38
98.17	Roditis		-	23.4 ± 0.63	23.4 ± 0.12	20.7 ± 0.23
102.1	Geisenheim 26		-	-	35.0 ± 0.30	23.8 ± 0.24
102.2	Geisenheim 26		-	21.9 ± 0.05	22.2 ± 0.32	21.9 ± 0.25
102.3	Chardonnay		-	18.6 ± 0.32	19.7 ± 0.14	24.6 ± 0.21
102.4	Chardonnay		-	22.6 ± 0.63	22.9 ± 0.27	21.0 ± 0.19
102.5	Pinot Noir	Germany	-	20.8 ± 0.35	19.6 ± 0.21	20.1 ± 0.11
102.6	Pinot Noir		-	17.6 ± 0.16	19.7 ± 0.11	20.4 ± 0.16
102.7	Pinot Noir		-	14.1 ± 0.24	18.2 ± 0.24	21.5 ± 0.18
102.8	Pinot Noir		-	16.7 ± 0.36	21.9 ± 0.26	20.8 ± 0.20
102.9	Pinot Blanc		-	-	34.8 ± 0.21	21.1 ± 0.17

102.10	Pinot Blanc	-	-	33.9 ± 0.14	22.3 ± 0.13
102.11	Riesling	-	-	-	24.1 ± 0.15
102.12	Riesling	+	20.4 ± 0.25	16.6 ± 0.31	21.3 ± 0.16
102.13	Riesling	+	21.2 ± 0.13	21.3 ± 0.45	22.1 ± 0.19
102.14	Riesling	-	20.4 ± 0.9	20.3 ± 0.21	21.6 ± 0.13
102.15	Riesling	-	22.5 ± 0.41	33.7 ± 0.16	23.5 ± 0.18
102.16	Riesling	-	18.5 ± 0.68	18.6 ± 0.47	25.1 ± 0.21
102.17	Riesling	-	19.1 ± 0.13	18.8 ± 0.31	23.5 ± 0.12
102.18	Riesling	-	26.6 ± 0.46	23.7 ± 0.45	24.3 ± 0.35
102.19	Riesling	-	21.6 ± 0.28	18.3 ± 0.36	21.8 ± 0.33
102.20	Riesling	+	23.4 ± 0.51	19.4 ± 0.12	23.1 ± 0.29

*****The infected status of these samples was assessed by ELISA in most cases, except for: LR-1, tested by RT-PCR (Osman *et al.*, 2007); Marsaoui, tested by HTS; 91.1 to 91.12, tested by RT-PCR (Komínek et al., 2005. *Virus Genes* 31, 247–255).
