# Supplementary material for: A Novel and Highly Inclusive Quantitative Real-Time RT-PCR Method for the Broad and Efficient Detection of Grapevine Leafroll-Associated Virus 1
Source: Plants (Basel). 2023 Feb 15;12(4):876. doi: 10.3390/plants12040876 (PMC9962094; doi:10.3390/plants12040876)
Supplement: Supplementary file 1 [file plants-12-00876-s001.zip › Table S2.pdf]

Table S2. Analytical sensitivity evaluation and absolute quantification of ten-fold serial dilutions of GLRaV-1 infected plant extracts.

**Dilutions of GLRaV-1 infected plant extracts**	**New real-time RT-PCR protocol**	
	**Average CTs ± SE**	**GLRaV-1 quantification (copies/µl)**
Undiluted extract	14.3 ± 0.23	5.8×10^7
1:10	16.84 ± 0.56	7.7×10^6
1:100	20.57 ± 0.22	4.5×10^5
1:1000	23.68 ± 0.39	5.3×10^4
1:10.000	26.81 ± 0.34	6.1×10^3
1:100.000	29.81 ± 0.28	7.6×10^2
1:1.000.000	33.43 ± 0.56	6.7 x 10
Healthy plant	-	-
